# The relationships between multimorbidity, depressive symptoms, health service utilization, and activities of daily living among the elderly in China

**DOI:** 10.1371/journal.pone.0333923

**Published:** 2025-10-09

**Authors:** Lingqian Bao, Wenyun Zhang, Yudie Zhang, Min Wang, Ying Chen, Xiao Shan, Yubo Xing

**Affiliations:** 1 Department of Nursing, Hangzhou Normal University, Hangzhou, Zhejiang, China; 2 Department of Otolaryngology Head and Neck Surgery, The Affiliated Hospital of Hangzhou Normal University, Hangzhou, Zhejiang, China; University of Oxford, UNITED KINGDOM OF GREAT BRITAIN AND NORTHERN IRELAND

## Abstract

**Objective:**

This study aimed to examine the relationships between multimorbidity, depressive symptoms, health service utilization (HSU), and activities of daily living (ADL) among elderly Chinese individuals using nationally representative data. It also explored the mediating roles of depressive symptoms and HSU in the association between multimorbidity and ADL impairment, to inform strategies for improving ADL function and quality of life among the elderly.

**Methods:**

The study utilized data from the 2020 wave of the China Health and Retirement Longitudinal Study (CHARLS), including 10,631 individuals aged 60 and above. A binary logistic regression model was applied to identify risk factors associated with ADL impairment in this population. Additionally, the mediating effects of depressive symptoms and HSU on the link between multimorbidity and ADL impairment were assessed using the Bootstrap method.

**Results:**

In 2020, 66.2% of elderly Chinese reported multimorbidity, 43.1% experienced depressive symptoms, and 30.0% had ADL impairment. Binary logistic regression identified advanced age, female gender, recent hospitalization, multimorbidity, and depressive symptoms as independent risk factors for ADL impairment, while higher education, better self-rated health, and health insurance coverage served as protective factors. Mediation analysis showed that depressive symptoms and HSU partially mediated the link between multimorbidity and ADL impairment, accounting for 39.64% (depressive symptoms), 2.98% (outpatient visits), and 11.79% (hospitalization) of the total effect.

**Conclusions:**

This study found that multimorbidity, depressive symptoms, and HSU significantly influence ADL function among elderly Chinese adults, with depressive symptoms showing a strong mediating effect. The findings highlight the need to manage multimorbidity, address mental health, and optimize healthcare use to enhance geriatric care and quality of life.

## Introduction

In recent years, the global prevalence of multimorbidity has steadily increased. Multimorbidity is defined as the coexistence of two or more chronic diseases within an individual [1]. A systematic review and meta-analysis across 54 countries, involving nearly 15.4 million people, found that 51.0% of individuals aged 60 and above worldwide suffer from multimorbidity [2]. In the United States, the prevalence of multimorbidity among the elderly is reported at 36.5% [3]. In Europe, it is projected that multimorbidity prevalence in the elderly will rise from 45.7% in 2015 to 52.8% by 2035 [4]. As one of the countries experiencing rapid population aging, China faces an even more severe burden of multimorbidity among its elderly population. According to Li et al. [5], the prevalence of multimorbidity among Chinese elderly individuals has reached 65.14%.

Activities of daily living (ADL) refer to the fundamental tasks individuals perform daily to maintain independent living [6]. These activities are essential for preserving autonomy and overall health in the elderly. ADL impairment negatively affects both physical and mental health and places a substantial caregiving burden on families and society [7]. In China, with its large aging population, the prevalence of ADL impairment among those aged 65 and above is notably high, ranging from 20% to 40% [8]. Research indicates that elderly individuals with multimorbidity are more likely to experience ADL impairment. A cohort study in Norway showed that elderly people with multimorbidity have a 24% higher risk of ADL impairment compared to those without multimorbidity [9].

Mental health is a critical component of elderly quality of life, with depressive symptoms being prevalent and posing a significant public health challenge [10]. Studies have demonstrated a bidirectional relationship between depressive symptoms and ADL impairment in the elderly [11]. Depressive symptoms may lead to ADL impairment by affecting cognitive function [12]. Conversely, ADL impairment restricts daily activities and social interactions, diminishing self-efficacy, increasing loneliness, lowering quality of life, and thereby exacerbating depressive symptoms [13]. Sleep disturbance, a common health issue among the elderly, is closely linked to depressive symptoms. For example, in patients with pituitary adenomas, depressive symptoms indirectly influence fatigue through sleep disturbance [14]. Patients with sarcopenia often experience worsened depressive symptoms due to decreased physical function. Sarcopenia not only impairs mobility but may also exacerbate depressive symptoms by increasing feelings of loneliness and helplessness. A longitudinal study found that elderly individuals with sarcopenia are more likely to develop new-onset depressive symptoms [15]. These findings highlight the complex interactions between physiological conditions and mental health in the elderly.

Health service utilization (HSU) is a key aspect of health management and service delivery for older populations and is closely related to multimorbidity, ADL impairment, and depressive symptoms. Compared to individuals with a single chronic disease, those with multimorbidity generally have poorer quality of life [16], as well as higher risks of disability and mortality, leading to increased demand for health services. Elderly individuals with ADL impairment may face difficulties in accessing health services. Furthermore, frequent HSU may worsen their ADL impairment [17]. Elderly individuals experiencing depressive symptoms may be more willing to seek health services due to physical discomfort, in contrast to those with better emotional health [18].

Previous research has primarily focused on the relationships between multimorbidity, ADL impairment, and depressive symptoms, or the interactions among these factors. These studies have revealed a strong association between multimorbidity and ADL impairment and highlighted that depressive symptoms can exacerbate functional disability in the elderly [19,20]. However, the role of HSU as a potential mediator in this relationship has received limited attention. Elderly individuals often increase their use of health services when facing multimorbidity and ADL impairment, which may significantly impact their overall health. Notably, HSU tends to be higher among those with depressive symptoms, potentially further influencing their ADL capabilities. To date, no studies have examined how multimorbidity affects ADL impairment through depressive symptoms and HSU in the elderly population of China.

Using data from the China Health and Retirement Longitudinal Study (CHARLS), this study aims to investigate the complex relationships between multimorbidity, depressive symptoms, HSU, and ADL impairment. We hypothesize that depressive symptoms and HSU mediate the relationship between multimorbidity and ADL impairment, ultimately influencing the functional status of the elderly. The findings will provide theoretical insights for managing elderly health, optimizing chronic disease healthcare systems, and enhancing the overall well-being of the elderly population.

## Methods

### Designated population and sample

The data for this study were obtained from the fifth wave of the CHARLS conducted in 2020. CHARLS employs a stratified, multi-stage probability sampling method proportional to population size to collect high-quality microdata representative of individuals aged 45 and older in China [21]. This dataset is specifically designed to analyze the challenges posed by population aging in China. In 2020, CHARLS covered 150 county-level units and 450 village-level units, including 19,395 individuals from approximately 12,400 households. The CHARLS dataset is publicly available for access via the official website, and registered users can download and use it (https://charls.charlsdata.com/pages/data/111/zh-cn.html). After excluding respondents with missing data on multimorbidity and ADL, the final sample comprised 10,631 individuals. Remaining missing data were addressed using the Chained Random Forests multiple imputation method, which predicts and updates missing values through initial imputation, determines the imputation order, and iteratively constructs random forest models [22]. The imputed results were combined to yield more reliable statistical analyses. This imputation process can be implemented using the *mice* package in R.

### Ethics approval

The CHARLS study received approval from the Peking University Biomedical Ethics Committee (IRB00001052–11015). All participants provided informed consent prior to their involvement in the study.

### Research variables

#### Multimorbidity.

In the 2020 CHARLS, 15 types of chronic diseases were investigated. This study focused on 12 self-reported physical chronic conditions: hypertension, dyslipidemia, diabetes, malignant tumors, chronic lung disease, liver disease, heart disease, stroke, kidney disease, asthma, stomach or digestive system diseases, and arthritis/rheumatism. To minimize potential recall bias, individuals who self-reported emotional or mental disorders, memory-related diseases, or Parkinson’s disease were excluded. Emotional or psychiatric disorders and memory impairments can affect patients’ self-awareness and reporting accuracy. Moreover, Parkinson’s disease is often accompanied by cognitive and emotional challenges, in addition to motor impairments, which could introduce bias or confound the results. To maintain data accuracy and study objectivity, these individuals were excluded. Participants reporting two or more chronic conditions were classified as having multimorbidity; otherwise, they were considered as having no multimorbidity.

#### ADL.

The ability to perform ADL was assessed using the Katz Index of Independence in ADL, which evaluates six tasks: feeding, dressing, transferring, toileting, bathing, and continence [23]. Each task is scored on a scale from 1 to 4, ranging from “no difficulty” to “unable to complete,” resulting in a total score between 6 and 24. Higher scores indicate greater functional impairment. In this study, difficulty with any one of the six activities was classified as physical functional impairment. Participants with such impairment were coded as 1, while those without impairment were coded as 0.

#### HSU.

HSU was evaluated based on the frequency of outpatient visits in the past month and inpatient visits in the past year. Respondents were asked, “How many times have you visited a healthcare facility or received in-home medical services in the past month?” and “How many times have you been hospitalized in the past year?” A frequency of one or more visits was coded as 1, while zero visits were coded as 0.

### Depressive symptoms

Depressive symptoms were assessed using the 10-item Center for Epidemiologic Studies Depression Scale (CES-D) [24]. The items include: (1) I was bothered by things that usually don’t bother me; (2) I had trouble keeping my mind on what I was doing; (3) I felt depressed; (4) I felt that everything I did was an effort; (5) I felt hopeful about the future; (6) I felt fearful; (7) My sleep was restless; (8) I was happy; (9) I felt lonely; and (10) I could not get “going.” Each item is rated on a 4-point scale: rarely or none of the time (<1 day), some or a little of the time (1–2 days), occasionally or a moderate amount of time (3–4 days), and most or all of the time (5–7 days). Scores for items 5 and 8 are reverse-coded. The total score ranges from 0 to 30, with higher scores indicating more severe depressive symptoms. A score of 10 or above indicates the presence of depressive symptoms.

### Sociodemographic characteristics

This study included demographic variables as control factors: gender (male, female), age groups (60–69, 70–79, ≥ 80 years), education level (illiterate, primary school, middle school, high school and above), marital status (married, unmarried), place of residence (urban, rural), annual household income (no income, 0–10,000, 10,001–20,000, > 20,000 CNY), self-rated health (good, average, poor), and health insurance status (none, Urban Employee Medical Insurance [UEMI], Urban-Rural Resident Medical Insurance [URRMI], others).

### Statistical analysis

Statistical analyses were performed using SPSS version 25.0. Categorical variables were presented as percentages, and chi-square tests were used to examine differences in ADL impairment across demographic groups. A binary logistic regression model identified risk factors for ADL impairment among the elderly. The mediating effects of depressive symptoms and HSU on the relationship between multimorbidity and ADL impairment were evaluated using Model 4 of the PROCESS 4.0 macro. Significance was determined via bootstrapping with 5,000 samples to estimate confidence intervals for indirect, direct, and total effects. All tests were two-tailed, with a significance level of α = 0.05.

## Results

### Sociodemographic data and ADL status

Among the 10,631 elderly participants, the majority were aged 60–69 years (61.1%), female (51.6%), married (76.8%), illiterate (53.8%), living in rural areas (65.8%), and had an annual household income below 20,000 RMB (73.7%). Half of the participants rated their health as average (50.3%), and 94.6% had medical insurance coverage. In the past month, 21.9% had outpatient visits, and 24.0% had been hospitalized within the past year. Additionally, 43.1% exhibited depressive symptoms, 30.0% experienced ADL impairment, and the prevalence of multimorbidity was 66.2%. As shown in Table 1, all sociodemographic variables, multimorbidity, depressive symptoms, and the frequencies of outpatient visits and hospitalizations were significantly associated with ADL impairment (**P* *< 0.001) (S1 Table).

**Table 1:** Sociodemographic characteristics and ADL status of all respondents.

Classification	Total *N* (%)	Disability Status *N* (%)		Χ^2^	P
All participants	10631(100)	Non-disabled 446(70.0)	Disabled 3185(30.0)		
**Age/years**				264.376	<0.001
60-69	6499(61.1)	4887(75.2)	1612(24.8)		
70-79	3142(29.6)	2037(64.8)	1105(35.2)		
≥80	990(9.3)	522(52.7)	468(47.3)		
**Gender**				135.567	<0.001
Male	5143(48.4)	3877(75.4)	1266(24.6)		
Female	5488(51.6)	3569(65.0)	1919(35.0)		
**Marriage status**				76.946	<0.001
Married	8164(76.8)	5893(72.2)	2271(27.8)		
Unmarried	2467(23.2)	1553(63.0)	914(37.0)		
**Education**				220.450	<0.001
Illiterate	5723(53.8)	3686(64.4)	2037(35.6)		
Primary school	2197(20.7)	1593(72.5)	604(27.5)		
Secondary school	1720(16.2)	1363(79.2)	357(20.8)		
High school and above	991(9.3)	804(81.1)	187(18.9)		
**Residence**				73.025	<0.001
Rural	6998(65.8)	4710(67.3)	2288(32.7)		
Urban	3633(34.2)	2736(75.3)	897(24.7)		
**Annual household income/RMB**				63.202	<0.001
No income	3682(34.6)	2559(69.5)	1123(30.5)		
1-10,000	3105(29.2)	2053(66.1)	1052(33.9)		
10,001-20,000	1050(9.9)	725(69.0)	325(31.0)		
>20,000	2794(26.3)	2109(75.5)	685(24.5)		
**Self-rated health**				1296.467	<0.001
Good	2158(20.3)	1851(85.8)	307(14.2)		
Average	5349(50.3)	4168(77.9)	1181(22.1)		
Poor	3124(29.4)	1427(45.7)	1697(54.3)		
**Health insurance**				99.526	<0.001
None	572(5.4)	353(61.7)	219(38.3)		
UEMI	2027(19.1)	1589(78.4)	438(21.6)		
URRMI	7718(72.6)	5272(68.3)	2446(31.7)		
Others	314(2.9)	232(73.9)	82(26.1)		
**Outpatient visits**				134.126	<0.001
0 visits	8308(78.1)	6045(72.8)	2263(27.2)		
≥1 visit	2323(21.9)	1401(60.3)	922(39.7)		
**Hospitalizations**				305.143	<0.001
0 visits	8076(76.0)	6009(74.4)	2067(25.6)		
≥1 visit	2555(24.0)	1437(56.2)	1118(43.8)		
**Multimorbidity**				443.409	<0.001
Yes	7038(66.2)	4459(63.4)	2579(36.6)		
No	3593(33.8)	2987(83.1)	606(16.9)		
**Depressive symptoms**				879.983	<0.001
Yes	4577(43.1)	2512(54.9)	2065(45.1)		
No	6054(56.9)	4934(81.5)	1120(18.5)		

Abbreviations: UEMI: Urban Employee Medical Insurance; URRMI: Urban-Rural Resident Medical Insurance.

### Binary logistic regression analysis of ADL impairment

A binary logistic regression analysis was performed with disability status as the dependent variable and sociodemographic characteristics, outpatient visits, hospitalizations, multimorbidity, and depressive symptoms as independent variables. The results in Table 2 showed that older age, female gender, hospitalization in the past year, multimorbidity, and depressive symptoms were significant risk factors for ADL impairment among the elderly. In contrast, higher education levels, better self-rated health (good or average) and having health insurance coverage were protective factors. Outpatient visits in the past month were not significantly associated with ADL impairment (*P* > 0.05) (S2 Table).

**Table 2:** Binary logistic regression results.

Variable	B	SE	Waldχ2	*OR* (95%*CI*)	*P*
**Age/year**	0.453	0.037	147.105	1.573(1.462-1.692)	<0.001
**Gender (Ref = Male)**					
**Female**	0.228	0.055	17.126	1.256(1.128-1.400)	<0.001
**Marriage status (Ref = Unmarried)**					
**Married**	0.003	0.059	0.003	1.003(0.849-1.126)	0.953
**Education**	−0.168	0.029	34.018	0.845(0.799−0.895)	<0.001
**Residence (Ref = Urban)**					
**Rural**	0.095	0.063	2.293	1.100(0.972-1.254)	0.130
**Annual household income/RMB**	−0.030	0.021	2.129	0.970(0.932-1.010)	0.145
**Self-rated health (Ref = Poor)**					
**Good**	−1.318	0.079	280.168	0.268(0.229-0.312)	<0.001
**Average**	−1.048	0.053	389.101	0.351(0.316-0.389)	
**Health insurance (Ref = None)**					
**UEMI**	−0.417	0.133	9.895	0.659(0.508-0.855)	0.002
**URRMI**	−0.203	0.101	4.007	0.817(0.670-0.996)	0.045
**Others**	−0.212	0.177	1.441	0.809(0.572-1.144)	0.230
**Outpatient visits (Ref = None)**	0.107	0.057	3.552	1.113(0.996-1.243)	0.059
**Hospitalizations (Ref = None)**	0.366	0.055	44.711	1.442(1.295-1.605)	<0.001
**Multimorbidity (Ref = No)**					
**Yes**	0.589	0.058	104.245	1.802(1.609-2.017)	<0.001
**Depressive symptoms (Ref = No)**					
**Yes**	0.814	0.050	267.916	2.256(2.047-2.487)	<0.001

Ref refers to the control group.

### Mediating effect of depressive symptoms and HSU between multimorbidity and ADL

**Fig 1:**
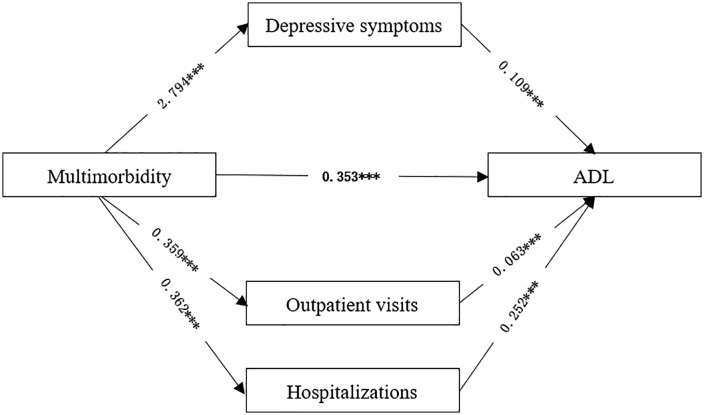
Path coefficient diagram for multimorbidity, depressive symptoms, HSU and ADL. ***P<0.001.

To investigate the underlying mechanism of the significant positive effect of multimorbidity on ADL impairment, depressive symptoms, outpatient visits, and hospitalizations were included as mediators in the structural equation model. The mediation effects were tested using Model 4 in the PROCESS macro for SPSS, and the indirect effects of depressive symptoms, outpatient visits, and hospitalizations on the relationship between multimorbidity and ADL impairment were confirmed through the Bootstrap method as described by Hayes [25] (Fig 1).

As shown in Table 3, the Bootstrap 95% confidence intervals (CIs) for the mediation effects of depressive symptoms and HSU on the relationship between multimorbidity and ADL did not include zero. This indicates that multimorbidity has both a direct effect on ADL impairment and an indirect effect mediated by depressive symptoms and HSU. Specifically, the indirect effect via depressive symptoms (0.306) accounted for 39.64% of the total effect (0.772). For HSU, the indirect effects of outpatient visits (0.023) and hospitalizations (0.091) accounted for 2.98% and 11.79%, respectively, of the total effect. Therefore, depressive symptoms and HSU partially mediate the relationship between multimorbidity and ADL impairment in the elderly (S3 Table).

**Table 3:** Mediation effect test of depressive symptoms and HSU between multimorbidity and ADL.

Effect Relationship		Effect	SE	LLCI	ULCI	Relative Effect
**Indirect effect**	multimorbidity→depressive symptoms→ADL	0.306	0.020	0.268	0.345	39.64%
	multimorbidity→outpatient visits→ADL	0.023	0.011	0.002	0.046	2.98%
	multimorbidity→hospitalizations→ADL	0.091	0.016	0.063	0.126	11.79%
**Direct effect**		0.353	0.051	0.254	0.452	45.60%
**Total effect**		0.772	0.052	0.671	0.873	100%

## Discussion

The health challenges faced by China’s elderly population are notably complex. Approximately 66.2% suffer from multimorbidity, 43.1% experience depressive symptoms, and 30.0% exhibit ADL impairment. Additionally, 24.0% were hospitalized in the past year, and 21.9% received outpatient care in the past month. These figures underscore the multifaceted difficulties in managing elderly health. Further analysis reveals that depressive symptoms and HSU partially mediate the link between multimorbidity and ADL impairment, suggesting that addressing mental health and improving healthcare accessibility may help reduce functional decline.

The binary logistic regression results on factors influencing ADL impairment align broadly with previous studies [26,27]. However, this study found no statistically significant association between outpatient visits in the past month and ADL impairment (*P* = 0.059). Although not meeting the conventional significance threshold, this borderline result suggests the need for further exploration. Prior research has indicated that individuals with metabolic multimorbidity tend to have increased outpatient visits and inpatient days, reflecting higher HSU [28]. We hypothesized that multimorbidity would lead to ADL impairment, thereby increasing outpatient and inpatient visits, but this study’s findings did not support that. Firstly, disabled elderly individuals often have complex health needs and may depend more on preventive and rehabilitative care rather than outpatient treatment alone. For instance, patients with physical limitations may receive insufficient rehabilitation during outpatient visits, limiting short-term improvement in ADL. Additionally, patients with acute conditions, such as cancer or cardiovascular disease, often require both outpatient and inpatient care, which may confound the relationship between ADL impairment and outpatient visits [29]. Secondly, the one-month observation period for outpatient visits may be too short to capture the gradual onset of ADL impairment. Future studies could extend this window or utilize longitudinal data to examine potential lag effects between ADL impairment and healthcare utilization over time. Moreover, community care and social support services may reduce disabled elderly individuals’ reliance on outpatient services. Further research is needed to understand how such supports, especially long-term and home care, contribute to improving ADL status. The WHO’s Integrated Care for Older People (ICOPE) model offers a promising framework to optimize health management for elderly individuals with ADL impairment. It promotes interdisciplinary collaboration to integrate diagnosis, treatment, care, rehabilitation, and health promotion, addressing the complex needs of the elderly [30]. Unlike short-term outpatient care, elderly patients with ADL impairment require sustained, continuous care. Multidisciplinary teams can develop personalized rehabilitation plans, combining geriatrics, rehabilitation, and community health resources to restore function and enhance quality of life. Therefore, future research should adopt longer observation periods or longitudinal designs to better capture the relationship between ADL impairment and HSU. Emphasis should also be placed on the impact of integrated medical and social support services, particularly long-term care, home care, and community support, on rehabilitation outcomes, which may reduce outpatient visits and improve health management for the elderly.

Additionally, this study found that depressive symptoms play a major mediating role between multimorbidity and ADL impairment, with an effect size of 0.306, accounting for 39.64% of the total effect. This suggests that multimorbidity increases psychological burden, leading to or worsening depressive symptoms, which in turn reduce the elderly’s ability to perform daily activities. Specifically, the chronic physical discomfort and social functioning decline caused by multimorbidity place significant psychological stress on older adults, potentially triggering depressive symptoms. These symptoms further impair their ability to manage basic tasks such as dressing, bathing, and eating. Previous studies often treated ADL as a mediator to explore how multimorbidity affects depressive symptoms, reporting indirect effects of 21.49% [31] and 32.52% [32]. In contrast, this study highlights the mediating role of depressive symptoms between multimorbidity and ADL, indicating that depressive symptoms are not merely an outcome of multimorbidity but also a critical factor influencing functional ability. This insight offers a new perspective on how multimorbidity impacts elderly daily functioning and underscores the importance of managing depressive symptoms in elderly care. Based on these findings, healthcare providers should prioritize the mental health of elderly patients with multimorbidity, regularly screening for and intervening in depressive symptoms to alleviate psychological distress and thereby improve ADL performance.

Moreover, this study also found that outpatient and inpatient visits partially mediate the relationship between multimorbidity and ADL, though their effects are smaller (2.98% and 11.79%, respectively). This suggests that HSU can help mitigate the negative impact of multimorbidity on daily functioning, reflecting the complex interplay between elderly health status and functional capacity. By exploring the indirect effects of multimorbidity through HSU, a relatively under-researched area, this study carries important public health implications. It highlights that elderly care should address not only the direct effects of multimorbidity but also optimize health service use to improve ADL. In conclusion, this study demonstrates that multimorbidity affects ADL in the elderly, with depressive symptoms, outpatient visits, and inpatient visits acting as three independent mediators. These findings provide empirical support for targeted elderly health management and offer a theoretical foundation for future intervention strategies.

## Conclusions

Based on the 2020 CHARLS data, this study found that multimorbidity, depressive symptoms, and HSU significantly influence ADL among the elderly in China. Depressive symptoms and HSU serve as important mediators between multimorbidity and ADL impairment, underscoring the need to address mental health and optimize healthcare use in elderly care. Future interventions should not only focus on managing multimorbidity but also prioritize treating depressive symptoms and improving health service accessibility. Policymakers are encouraged to develop a comprehensive health promotion framework that integrates physical, psychological, and social support, and to implement coordinated intervention strategies aimed at enhancing the daily functioning and overall quality of life of the elderly.

### Strengths and limitations

This study, based on a nationally representative sample, examined the relationships between multimorbidity, depressive symptoms, HSU, and ADL among the elderly in China. It identified the mediating roles of depressive symptoms and HSU in the link between multimorbidity and ADL impairment, offering valuable insights for elderly health management and policy development. However, several limitations should be noted. First, to minimize recall bias in self-reported multimorbidity data, individuals with cognitive impairment were excluded. While this improved data accuracy, it may have underestimated the health status of those with severe multimorbidity, especially with concurrent cognitive decline. Second, the cross-sectional design limits the ability to capture temporal changes and infer causality. Longitudinal studies are needed to explore these dynamic relationships over time. Finally, the analysis of HSU did not consider cost factors, which could affect the findings. Future research should incorporate cost considerations to improve accuracy.

## Supporting information

S1 Table Sociodemographic characteristics and ADL status of all respondents. (DOCX)

S2 Table Binary logistic regression results. (DOCX)

S3 Table Mediation effect test of depressive symptoms and HSU between multimorbidity and ADL. (DOCX)
