# Supplementary material for: The relationships between multimorbidity, depressive symptoms, health service utilization, and activities of daily living among the elderly in China
Source: PLoS One. 2025 Oct 9;20(10):e0333923. doi: 10.1371/journal.pone.0333923 (PMC12510492; doi:10.1371/journal.pone.0333923)
Supplement: S1 Table — (DOCX) [file pone.0333923.s001.docx]

**Table 1. Sociodemographic characteristics and ADL status of all respondents.**

| **Classification** | **Total**  ***N* (%)** | **Disability Status *N* (%)** | | **Χ^2^** | **P** |
| --- | --- | --- | --- | --- | --- |
| **All participants** | 10631(100) | **Non-disabled** 7446(70.0) | **Disabled**  3185(30.0) |  |  |
| **Age/years** |  |  |  | 264.376 | ＜0.001 |
| 60-69 | 6499(61.1) | 4887(75.2) | 1612(24.8) |  |  |
| 70-79 | 3142(29.6) | 2037(64.8) | 1105(35.2) |  |  |
| ≥80 | 990(9.3) | 522(52.7) | 468(47.3) |  |  |
| **Gender** |  |  |  | 135.567 | ＜0.001 |
| Male | 5143(48.4) | 3877(75.4) | 1266(24.6) |  |  |
| Female | 5488(51.6) | 3569(65.0) | 1919(35.0) |  |  |
| **Marriage status** |  |  |  | 76.946 | ＜0.001 |
| Married | 8164(76.8) | 5893(72.2) | 2271(27.8) |  |  |
| Unmarried | 2467(23.2) | 1553(63.0) | 914(37.0) |  |  |
| **Education** |  |  |  | 220.450 | ＜0.001 |
| Illiterate | 5723(53.8) | 3686(64.4) | 2037(35.6) |  |  |
| Primary school | 2197(20.7) | 1593(72.5) | 604(27.5) |  |  |
| Secondary school | 1720(16.2) | 1363(79.2) | 357(20.8) |  |  |
| High school and above | 991(9.3) | 804(81.1) | 187(18.9) |  |  |
| **Residence** |  |  |  | 73.025 | ＜0.001 |
| Rural | 6998(65.8) | 4710(67.3) | 2288(32.7) |  |  |
| Urban | 3633(34.2) | 2736(75.3) | 897(24.7) |  |  |
| **Annual household income/RMB** |  |  |  | 63.202 | ＜0.001 |
| No income | 3682(34.6) | 2559(69.5) | 1123(30.5) |  |  |
| 1-10,000 | 3105(29.2) | 2053(66.1) | 1052(33.9) |  |  |
| 10,001-20,000 | 1050(9.9) | 725(69.0) | 325(31.0) |  |  |
| ＞20,000 | 2794(26.3) | 2109(75.5) | 685(24.5) |  |  |
| **Self-rated health** |  |  |  | 1296.467 | ＜0.001 |
| Good | 2158(20.3) | 1851(85.8) | 307(14.2) |  |  |
| Average | 5349(50.3) | 4168(77.9) | 1181(22.1) |  |  |
| Poor | 3124(29.4) | 1427(45.7) | 1697(54.3) |  |  |
| **Health insurance** |  |  |  | 99.526 | ＜0.001 |
| None | 572(5.4) | 353(61.7) | 219(38.3) |  |  |
| UEMI | 2027(19.1) | 1589(78.4) | 438(21.6) |  |  |
| URRMI | 7718(72.6) | 5272(68.3) | 2446(31.7) |  |  |
| Others | 314(2.9) | 232(73.9) | 82(26.1) |  |  |
| **Outpatient visits** |  |  |  | 134.126 | ＜0.001 |
| 0 visits | 8308(78.1) | 6045(72.8) | 2263(27.2) |  |  |
| ≥1 visit | 2323(21.9) | 1401(60.3) | 922(39.7) |  |  |
| **Hospitalizations** |  |  |  | 305.143 | ＜0.001 |
| 0 visits | 8076(76.0) | 6009(74.4) | 2067(25.6) |  |  |
| ≥1 visit | 2555(24.0) | 1437(56.2) | 1118(43.8) |  |  |
| **Multimorbidity** |  |  |  | 443.409 | ＜0.001 |
| Yes | 7038(66.2) | 4459(63.4) | 2579(36.6) |  |  |
| No | 3593(33.8) | 2987(83.1) | 606(16.9) |  |  |
| **Depressive symptoms** |  |  |  | 879.983 | ＜0.001 |
| Yes | 4577(43.1) | 2512(54.9) | 2065(45.1) |  |  |
| No | 6054(56.9) | 4934(81.5) | 1120(18.5) |  |  |

Abbreviations: UEMI: Urban Employee Medical Insurance; URRMI: Urban-Rural Resident Medical Insurance.
