# Supplementary material for: The relationships between multimorbidity, depressive symptoms, health service utilization, and activities of daily living among the elderly in China
Source: PLoS One. 2025 Oct 9;20(10):e0333923. doi: 10.1371/journal.pone.0333923 (PMC12510492; doi:10.1371/journal.pone.0333923)
Supplement: S2 Table — (DOCX) [file pone.0333923.s002.docx]

**Table 2. Binary logistic regression results.**

| **Variable** | **B** | | **SE** | **Waldχ2** | ***OR* (95%*CI*)** | ***P*** |
| --- | --- | --- | --- | --- | --- | --- |
| **Age/year** | 0.453 | 0.037 | | 147.105 | 1.573(1.462-1.692) | ＜0.001 |
| **Gender (Ref=Male)** |  |  | |  |  |  |
| **Female** | 0.228 | 0.055 | | 17.126 | 1.256(1.128-1.400) | ＜0.001 |
| **Marriage status (Ref=Unmarried)** |  | |  |  |  |  |
| **Married** | 0.003 | | 0.059 | 0.003 | 1.003(0.849-1.126) | 0.953 |
| **Education** | -0.168 | | 0.029 | 34.018 | 0.845(0.799-0.895) | ＜0.001 |
| **Residence (Ref=Urban)** |  | |  |  |  |  |
| **Rural** | 0.095 | | 0.063 | 2.293 | 1.100(0.972-1.254) | 0.130 |
| **Annual household income/RMB** | -0.030 | | 0.021 | 2.129 | 0.970(0.932-1.010) | 0.145 |
| **Self-rated health (Ref=Poor)** |  | |  |  |  |  |
| **Good** | -1.318 | | 0.079 | 280.168 | 0.268(0.229-0.312) | ＜0.001 |
| **Average** | -1.048 | | 0.053 | 389.101 | 0.351(0.316-0.389) |  |
| **Health insurance (Ref=None)** |  | |  |  |  |  |
| **UEMI** | -0.417 | | 0.133 | 9.895 | 0.659(0.508-0.855) | 0.002 |
| **URRMI** | -0.203 | | 0.101 | 4.007 | 0.817(0.670-0.996) | 0.045 |
| **Others** | -0.212 | | 0.177 | 1.441 | 0.809(0.572-1.144) | 0.230 |
| **Outpatient visits (Ref=None)** | 0.107 | | 0.057 | 3.552 | 1.113(0.996-1.243) | 0.059 |
| **Hospitalizations (Ref=None)** | 0.366 | | 0.055 | 44.711 | 1.442(1.295-1.605) | ＜0.001 |
| **Multimorbidity (Ref=No)** |  | |  |  |  |  |
| **Yes** | 0.589 | | 0.058 | 104.245 | 1.802(1.609-2.017) | ＜0.001 |
| **Depressive symptoms (Ref=No)** |  | |  |  |  |  |
| **Yes** | 0.814 | | 0.050 | 267.916 | 2.256(2.047-2.487) | ＜0.001 |

Ref refers to the control group.
