# Supplementary material for: The relationships between multimorbidity, depressive symptoms, health service utilization, and activities of daily living among the elderly in China
Source: PLoS One. 2025 Oct 9;20(10):e0333923. doi: 10.1371/journal.pone.0333923 (PMC12510492; doi:10.1371/journal.pone.0333923)
Supplement: S3 Table — (DOCX) [file pone.0333923.s003.docx]

**Table 3. Mediation effect test of depressive symptoms and HSU between multimorbidity and ADL.**

| **Effect Relationship** | | **Effect** | **SE** | **LLCI** | **ULCI** | **Relative Effect** |
| --- | --- | --- | --- | --- | --- | --- |
| **Indirect effect** | multimorbidity→depressive symptoms→ADL | 0.306 | 0.020 | 0.268 | 0.345 | 39.64% |
|  | multimorbidity→outpatient visits→ADL | 0.023 | 0.011 | 0.002 | 0.046 | 2.98% |
|  | multimorbidity→hospitalizations→ADL | 0.091 | 0.016 | 0.063 | 0.126 | 11.79% |
| **Direct effect** | | 0.353 | 0.051 | 0.254 | 0.452 | 45.60% |
| **Total effect** | | 0.772 | 0.052 | 0.671 | 0.873 | 100% |
